# Supplementary material for: Modelling Skylarks (Alauda arvensis) to Predict Impacts of Changes in Land Management and Policy: Development and Testing of an Agent-Based Model
Source: PLoS One. 2013 Jun 6;8(6):e65803. doi: 10.1371/journal.pone.0065803 (PMC3675089; doi:10.1371/journal.pone.0065803)
Supplement: Supporting Information S4 — The skylark ODdox as a zipped archive. (ZIP) [file pone.0065803.s004.zip › Skylark_ODdox/class_track.html]

ALMaSS Skylark ODdox: Track Class Reference


ALMaSS Skylark ODdox
 2.0


- Main Page
- Related Pages
- Classes
- Files

- Class List
- Class Index
- Class Hierarchy
- Class Members

Public Member Functions

Track Class Reference

`#include <elements.h>`

List of all members.

|  |  |
| --- | --- |
| Public Member Functions | |
|  | Track (void) |
| Public Member Functions inherited from NonVegElement | |
|  | NonVegElement (void) |
| Public Member Functions inherited from LE | |
| void | AddArea (double a\_area\_diff) |
| void | BumpRunNum (void) |
| virtual void | DoDevelopment (void) |
| virtual void | ForceGrowthDevelopment (void) |
| virtual void | ForceGrowthInitialize (void) |
| virtual void | ForceGrowthTest (void) |
| double | GetArea (void) |
| LE \* | GetBorder (void) |
| bool | GetCattleGrazing (void) |
| virtual int | GetCentroidX () |
| virtual int | GetCentroidY () |
| int | GetCountryDesignation (void) |
| virtual double | GetDayDegrees (void) |
| virtual double | GetDeadBiomass (void) |
| virtual double | GetDigestability (void) |
| virtual TTypesOfLandscapeElement | GetElementType (void) |
| int | GetFileType (void) |
| virtual double | GetGreenBiomass (void) |
| bool | GetHigh (void) |
| virtual double | GetInsectPop (void) |
| virtual double | GetLAGreen (void) |
| int | GetLastTreatment (int \*a\_index) |
| virtual double | GetLATotal (void) |
| int | GetMapIndex (void) |
| bool | GetMapValid (void) |
| int | GetMaxX (void) |
| int | GetMaxY (void) |
| int | GetMConstants (int a) |
| int | GetMDates (int a, int b) |
| long | GetMgtLoopDetectCount (void) |
| long | GetMgtLoopDetectDate (void) |
| int | GetMinX (void) |
| int | GetMinY (void) |
| long | GetOldDays (void) |
| Farm \* | GetOwner (void) |
| int | GetOwnerFile (void) |
| int | GetOwnerIndex (void) |
| int | GetPesticideCell () |
| bool | GetPigGrazing (void) |
| bool | GetPoison (void) |
| int | GetPoly (void) |
| int | GetRotIndex (void) |
| long | GetRunNum (void) |
| LE\_Signal | GetSignal (void) |
| virtual bool | GetSkScrapes (void) |
| int | GetSoilType () |
| int | GetSubType (void) |
| virtual double | GetTrafficLoad (void) |
| int | GetUnsprayedMarginPolyRef (void) |
| int | GetValidX (void) |
| int | GetValidY (void) |
| int | GetVegAge () |
| virtual double | GetVegBiomass (void) |
| virtual double | GetVegCover (void) |
| virtual int | GetVegDensity (void) |
| virtual double | GetVegHeight (void) |
| virtual bool | GetVegPatchy (void) |
| int | GetVegStore (void) |
| virtual TTypesOfVegetation | GetVegType (void) |
| virtual double | GetWeedBiomass (void) |
| bool | HasTramlines (void) |
| virtual void | Insecticide (double) |
| virtual void | InsectMortality (double) |
| bool | IsRecentlyMown (void) |
| int | IsRecentlySprayed (void) |
|  | LE (void) |
| virtual void | RecalculateBugsNStuff (void) |
| virtual void | ReduceVeg (double) |
| virtual void | ReduceVeg\_Extended (double) |
| virtual void | ReduceWeedBiomass (double) |
| void | ResetTrace (void) |
| void | SetArea (double a\_area) |
| void | SetBorder (LE \*a\_border) |
| virtual void | SetCentroid (int x, int y) |
| void | SetCopyTreatment (int a\_treatment) |
| void | SetCountryDesignation (int a\_designation) |
| virtual void | SetCropData (double, double, double, TTypesOfVegetation, double, bool) |
| virtual void | SetCropDataAll (double, double, double, double, TTypesOfVegetation, double, double, bool, double, bool, double) |
| void | SetElementType (TTypesOfLandscapeElement a\_type) |
| void | SetFileType (int a\_file\_type) |
| virtual void | SetGrowthPhase (int) |
| void | SetHerbicideDelay (int a\_decaytime\_days) |
| void | SetHigh (bool a\_high) |
| virtual void | SetInsectPop (double) |
| void | SetLastTreatment (int a\_treatment) |
| void | SetMapIndex (int a\_map\_index) |
| void | SetMapValid (bool a\_valid) |
| void | SetMaxX (int x) |
| void | SetMaxY (int y) |
| void | SetMConstants (int a, int c) |
| void | SetMDates (int a, int b, int c) |
| void | SetMgtLoopDetectCount (long a\_num) |
| void | SetMgtLoopDetectDate (long a\_num) |
| void | SetMinX (int x) |
| void | SetMinY (int y) |
| void | SetMownDecay (int a\_decaytime\_days) |
| void | SetOldDays (long a\_days) |
| void | SetOwner (Farm \*a\_owner, int a\_owner\_num, int a\_owner\_index) |
| void | SetPesticideCell (int a\_cell) |
| void | SetPoison (bool a\_poison) |
| void | SetPoly (int a\_poly) |
| void | SetRotIndex (int a\_index) |
| void | SetSignal (LE\_Signal a\_signal) |
| void | SetSoilType (int a\_st) |
| void | SetSubType (int a\_subtype) |
| void | SetTramlinesDecay (int a\_decaytime\_days) |
| void | SetUnsprayedMarginPolyRef (int a\_unsprayedmargin) |
| void | SetValidXY (int a\_valid\_x, int a\_valid\_y) |
| virtual void | SetVegBiomass (int) |
| virtual void | SetVegHeight (double, double, double, double) |
| virtual void | SetVegPatchy (bool) |
| void | SetVegStore (int a\_veg) |
| virtual void | SetVegType (TTypesOfVegetation, TTypesOfVegetation) |
| virtual void | StoreLAItotal () |
| void | Tick (void) |
| virtual void | ToggleCattleGrazing (void) |
| virtual void | TogglePigGrazing (void) |
| void | Trace (int a\_value) |
| virtual void | ZeroVeg (void) |
| void | ZeroVegAge () |
| virtual | ~LE (void) |

---

## Constructor & Destructor Documentation

|  |  |  |  |  |  |
| --- | --- | --- | --- | --- | --- |
| Track::Track | ( | void |  | ) |  |

References LE::m\_type, and tole\_Track.

: NonVegElement() {

m\_type = tole\_Track;

}

---

The documentation for this class was generated from the following files:

- elements.h
- elements.cpp


- Track
- Generated on Thu Jan 10 2013 13:15:38 for ALMaSS Skylark ODdox by
   1.8.1.1
